# Self-doped N, S porous carbon from semi-coking wastewater-based phenolic resin for supercapacitor electrodes

**DOI:** 10.3389/fchem.2022.1021394

**Published:** 2022-10-06

**Authors:** Long Yan, Xianjie Wang, Yufei Wang, Jian Li, Qianqian Liu, Xiang Zhong, Yuan Chang, Qingchao Li, Santosh Kumar Verma

**Affiliations:** ^1^ Shaanxi Key Laboratory of Low Metamorphic Coal Clean Utilization, School of Chemistry and Chemical Engineering, Yulin University, Yulin, China; ^2^ National Engineering Research Center of Coal Preparation and Purification, China University of Mining and Technology, Xuzhou, China

**Keywords:** semi-coking wastewater, porous carbon, supercapacitors, N-S doping, phenolic resin

## Abstract

Contamination of phenolic compounds has devastating effects on the environment. Therefore, its harmless treatment and recycling have received extensive attention. Herein, a novel method for preparing N-S doped phenolic resin (NSPR) from phenols, N and S groups in semi-coking wastewater, and formaldehyde are developed. The KOH is consequently incorporated into the NSPR through simultaneous carbonization and activation in a single step to produce porous carbon material (NSPC). The as-obtained NSPC exhibits a high specific capacitance of 182 F g^−1^ at 0.5 A g^−1^, a high energy density of 9.1 Wh kg^−1^ at a power density of 0.15 kW kg^−1^, and remarkable cycling stability in aqueous KOH electrolyte. This outstanding electrochemical performance is attributed to its ultrahigh specific surface area (SSA, 2,523 m^2^ g^−1^), enormous total pore volume (V_t_, 1.30 cm^3^ g^−1^), rational pore structure, and N-S heteroatom self-doping (0.76 at% N and 0.914 at% S), which ensures adequate charge storage, rapid electrolyte ion diffusion, and contributed pseudo-capacitance. This work not only provides a facile method for transforming phenolic wastewater into high-value products but also offers a cost-effective and high-performance porous carbon material for supercapacitors.

## Graphical Abstract

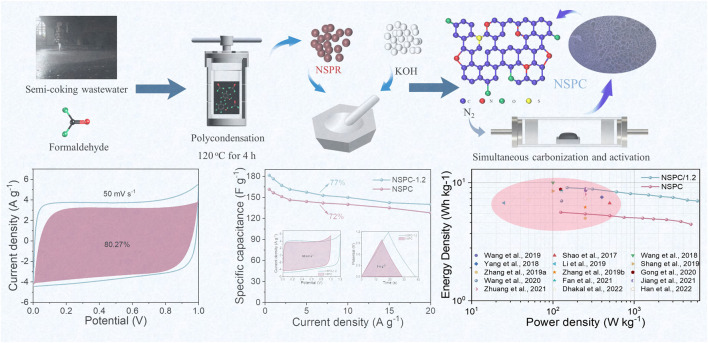

## Highlights

Direct utilization of phenolic wastewater to prepare phenolic resin.
N, S doped porous carbon was prepared by N and S elements in wastewater.
N, S doped porous carbon shows ultrahigh specific surface area (2523 m^2^ g^-1^).
Capacitance retention was 99% after 10,000 charge-discharge cycles.
Comparing the performance of supercapacitors at 1 V and 1.2 V voltage windows.

## Introduction

For decades, the coal industry has remained a major source of wastewater (Mz et al., 2020). In addition, numerous phenolic pollutants are present in coal chemical wastewater, which complicates its composition and water quality (Ji et al., 2015; He et al., 2021; Bao et al., 2022). Particularly, semi-coking wastewater (Liu et al., 2021), which is produced by the carbonization of coal at low (500–600°C) and medium (700–800°C) temperatures (Bai et al., 2022), is contaminated with phenols, ammonia, hydrogen sulfide, carbon dioxide, and oil, *etc.* It comprises numerous refractory and highly toxic pollutants, including benzene series, phenols, polycyclic aromatic hydrocarbons, nitrogen-oxygen, and sulfur-oxygen heterocyclic compounds, as well as inorganic pollutants such as heavy metals (Dargahi et al., 2021; Ding et al., 2021; Gao et al., 2022). The water quality of the wastewater is similar to that of coking wastewater, but the COD concentration of semi-coking wastewater is approximately 10 times higher, and the concentrations of ammonia nitrogen and phenols are also significantly higher than coking wastewater (Bokun et al., 2020). Semi-coking wastewater is the most harmful type of wastewater due to its high COD and high toxicity (Ma et al., 2017; Zhou et al., 2017; Li et al., 2020). Thus, the method of treating these wastewaters has always been the subject of intensive research (Wang et al., 2017; Maalouf and El-Fadel, 2020).

So far, semi-coking wastewater treatment technology is still in the exploratory stage, with no mature treatment process (Babuponnusami and Muthukumar, 2012). The majority of treatment processes are primarily based on the phenolic wastewater treatment method (Jiao et al., 2017). The activated sludge method is widely employed as one of the common methods for treating phenol-containing wastewater (Kamali et al., 2022). In contrast, the activated sludge process is not suitable and has relatively low efficiency in the treatment of high concentrations of phenol-containing wastewater (Anastasi et al., 2012). In addition, extraction is a common method for treating phenolic wastewater. However, the introduction of an extractant during extraction will lead to serious emulsification of wastewater, and the obtained products, particularly creosote substances, are difficult to separate, which severely hinders the implementation of the process (Zhang et al., 2020).

The prior studies primarily focused on the pretreatment of wastewater, particularly to degrade phenols and ammonia nitrogen compounds in wastewater, which not only wasted resources but also polluted the environment. The current method for treating semi-coking wastewater has several drawbacks, including a high investment cost, complicated operation, and substandard water quality upon discharge, making it unsuitable for industrial promotion (Liu et al., 2017). Therefore, an innovative method for converting phenolic wastewater from “treatment” to “conversion” is proposed, in which semi-coking wastewater rich in phenols and ammonia nitrogen is directly used to prepare N-S doped phenolic resin (NSPR), and the prepared NSPR is converted into porous carbon for supercapacitor. The advantages of the phenolic resin include a mature production process, repeatable performance of derived carbon, high carbonization yield, low impurity content, and ease of activation to form pores (Heimböckel et al., 2018; Ai et al., 2019; Yan et al., 2021). Several phenolic resin-based carbon materials for supercapacitor energy storage applications have been reported (Guo et al., 2019). For instance, Feng et al. prepared phenolic resins from thiourea and tetraethyl orthosilicate, then used the phenolic resins to prepare multi-heteroatom-doped carbon materials with a specific surface area (SSA) of 3,599.0 m^2^ g^−1^. The specific capacitance was 461.7 F g^−1^ at 0.1 A g^−1^ current density (Feng et al., 2022). Li et al. developed a cage-like lignin-based phenolic resin using a direct spray drying method. The resulting phenolic resin was carbonized with KOH to prepare porous carbon for supercapacitors (217.3 F g^−1^ at 0.5 A g^−1^) (Li et al., 2022). Dong et al. fabricated Fe-doped hierarchical porous carbons utilizing phenolic resin as the raw material and potassium ferrite (K_2_FeO_4_) as the catalyst and activator. The results revealed that the activated carbon materials exhibited a greater SSA of 1,086 m^2^ g^−1^. The specific capacitance of the activated phenolic resin was 315 F g^−1^ at 1 A g^−1^ (Dong et al., 2021). Therefore, the preparation of phenolic resin from semi-coking wastewater is presumed to be used in supercapacitor electrodes. Moreover, various chemical bond cleavage and rearrangement may occur during carbonization due to the complexity of the chemical composition and molecular structure in semi-coking wastewater; these factors promote the doping of heteroatoms in porous carbon (Wei et al., 2020). The electrochemical performance of supercapacitors based on porous carbon can be improved by structural defects and heteroatom self-doping from wastewater (Zou et al., 2018).

Currently, researchers usually use H_3_PO_4_, KOH, ZnCl_2_, and other activators (such as NaHCO_3_, FeCl_3_) to produce porous carbon (Peng et al., 2021a; Jiang et al., 2022). Among them, H_3_PO_4_ is an environmentally friendly activator that can function at rather low temperatures (about 500 C), resulting in mature mesoporous structures, but not microporous structures suitable for electrolyte ion storage. Although the carbon materials activated by ZnCl_2_, NaHCO_3_, and FeCl_3_ have high carbon yields, the cost is high, and the obtained carbon materials commonly have a well-developed microporous structure, rather than a mesoporous structure suitable for electrolyte penetration (Peng et al., 2021b). Compared with other activators, KOH can prevent the formation of tar, reduce the temperature of activation reaction, accelerate the removal of non-carbon components, and increase the rate of pyrolysis reaction (Gao et al., 2020). It is the most effective activator for preparing porous carbon with uniform pore size distribution. Therefore, we looked forward to activating NSPR using KOH as an activator.

In this study, refractory semi-coking wastewater was employed as the raw material to develop NSPR through a simple polycondensation reaction. Afterward, it was carbonized and activated in order to produce porous carbon for supercapacitor electrodes. The structure and composition of generated porous carbon (NSPC) from semi-coking wastewater were characterized, as well as the NSPC synthesis mechanism was investigated. The performances of an NSPC-based symmetrical supercapacitor with different voltages were also compared.

## Experimental

### Materials

The carbon source was semi-coking wastewater acquired from Yulin, Shaanxi, China. The content of phenols in semi-coking wastewater was determined by (National Standard, 2014), and the content was about 3,000 mg L^−1^ (Wang et al., 2020). The chemicals, including, formaldehyde, ethanol, acetone, potassium hydroxide, and hydrochloric acid, were purchased from Aladdin Industrial Inc., Shanghai, China. All chemicals were of analytical grade and utilized without further purification.

### Synthesis of carbon from semi-coking wastewater

**FIGURE 1:**
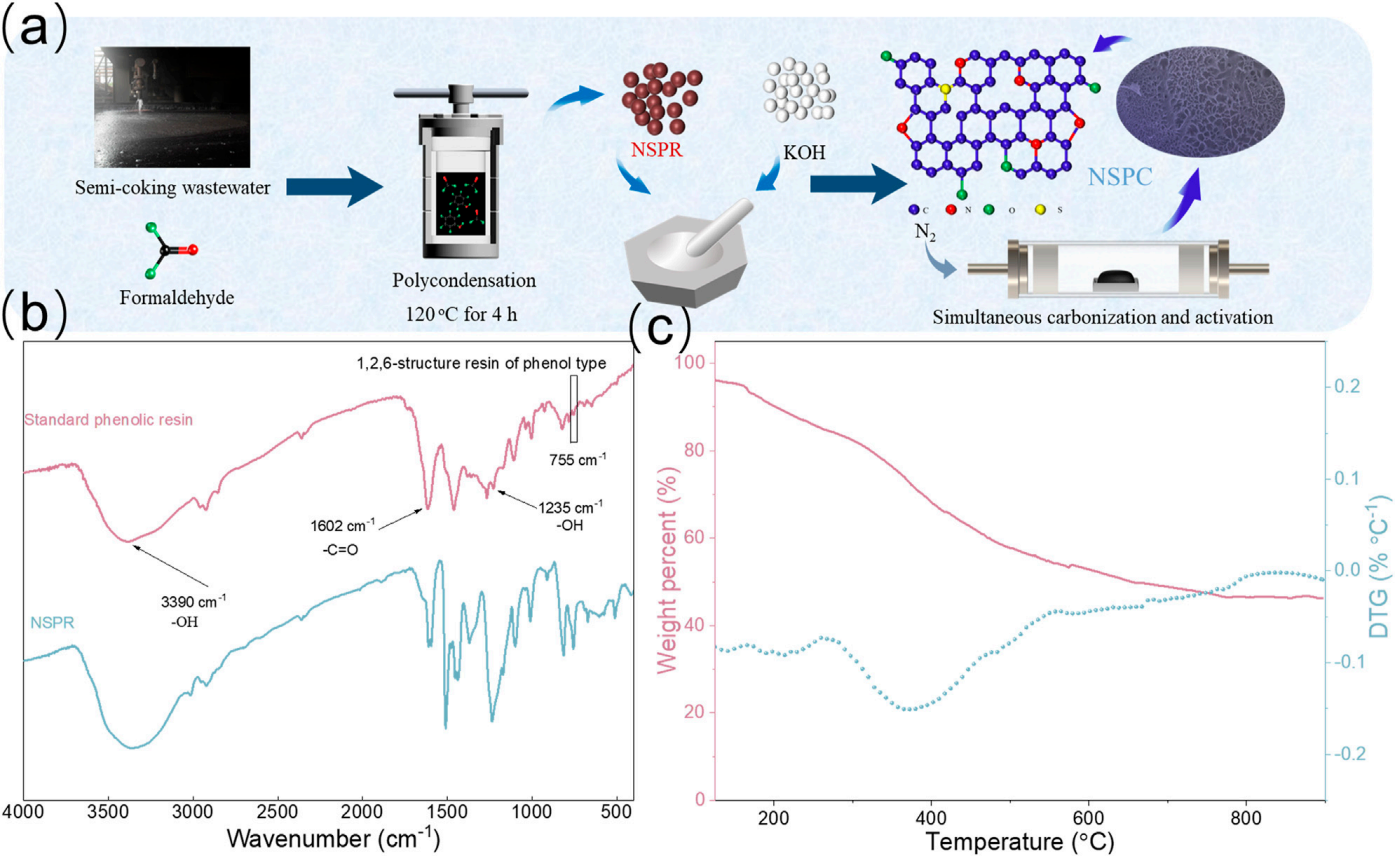
**(A)** Schematic diagram of the preparation process for NSPC, **(B)** FT-IR spectrum of NSPR, **(C)** TG/DTG curves.

The schematic illustration for NSPC synthesis from Semi-coking wastewater is depicted in Figure 1A. Initially, 80 ml Semi-coking wastewater and 1.4 mL formaldehyde were placed in a 100 ml Teflon-lined autoclave and heated to 120°C for 4 h. Then, the intermediate NSPR was obtained in an oven after 12 h of drying at 60°C. Next, the dried NSPR was ground into a powder in a mortar. Afterward, 3 g of NSPR powder and 9 g of KOH were mixed evenly in a mortar. The resulting mixture was carbonized and activated simultaneously in a tube furnace at 800°C for 1 h at a heating rate of 4°C min^−1^ under a nitrogen atmosphere. After cooling to room temperature, the activated material was washed with 1 M hydrochloric acid (HCl) and deionized water, then dried at 80°C for 24 h to yield N-S-doped porous material (NSPC). Comparatively, the NSPR powder was carbonized utilizing the same procedure but without KOH and labeled as NSC.

### Materials characterization

The microstructure and morphology of the as-prepared samples were examined by scanning electron microscopy (SEM, Zeiss, Sigma300) and high-resolution transmission electron microscopy (HRTEM, JEOL, JEM-F200). The XRD patterns of the samples were analyzed by X-ray diffraction spectroscope (XRD, Bruker D8 Advance, Cu Ka radiation). The elemental distributions of NSPC were confirmed using STEM (JEOL, JEM-ARM200F) and Energy-Dispersive X-ray (EDX) spectroscope. The Raman spectra of the samples were recorded using a Raman spectroscope (Horiba, LabRAM HR Evolution). The N_2_ adsorption-desorption isotherms were measured by using Quantachrome AUTOSORB-IQ3. The pore size distribution and the SSA were calculated using nonlocal density functional theory (NLDFT) and Brunauer-Emmett-Teller (BET) method, respectively. The X-ray photoelectron spectroscope (XPS, Thermo Kalpha) was employed to investigate the near-surface chemical species of materials utilizing monochromatic Al-Ka radiation. The elemental analysis of NSPR was performed using an elemental analyzer (Multi-EA5000). The thermal behavior of the intermediate NSPR was performed with a thermal analyzer (Hitachi, TG 7300). The sample was heated in an N_2_ atmosphere from 50 to 800°C at a rate of 5°C min^−1^. QP 2010W/0 RP230V gas chromatography-mass spectrometry (GC-MS, Shimadzu Co., Ltd., Japan) was used to analyze the main organics and their relative contents in the semi-coking wastewater.

### Electrochemical measurements

The working electrode was fabricated by dispersing NSPC, acetylene black, and PTFE in ethanol in a mass proportion of 8:1:1, and then smearing it onto nickel foam (1 cm^2^), which served as the current collector. The mass loading of NSPC on the working electrode was approximately 2.2–2.5 mg cm^−2^. Electrochemical characterization of NSPC was performed using the Electrochemical Workstation (CHI760E), including cyclic voltammetry (CV), galvanostatic charge-discharge (GCD), and electrochemical impedance spectroscopy (EIS).

The specific capacitance (*C*, F g^−1^) of a single working electrode for three-electrode and two-electrode system was calculated according to the GCD curves by Equation 1 and Equation 2, respectively.

C=I∆t/m∆V (1)

C=2I∆t/m∆V (2)

where *I* (A), *Δt* (s), *m* (g), and *ΔU* refers to the charge/discharge current, discharge time, mass loading of an electrode, and operating voltage excluding the voltage drop, respectively. The specific energy density *E* (Wh kg^−1^) and the power density *P* (W kg^−1^) of the device based on the total active material were estimated by Equation 3 and Equation 4.

$$E=C{\left(∆U\right)}^{2}/2\times 4\times 3.6$$ (3)

P=3600E/∆t (4)

The coulomb efficiency is calculated by Equation 5:

$$\eta =t_{d}/t_{c}$$ (5)

Where *t*_*d*_ (s) and *t*_*c*_ (s) are the discharge time and charge time, respectively.

## Results and discussion

### Characterization of NSPR

Figure 1A depicts the NSPC preparation process. NSPR was first synthesized from semi-coking wastewater and formaldehyde at 120 C. The FTIR patterns reveal that the NSPR has the same functional groups as in commercial phenolic resin (Figure 1B) (Han et al., 2017). The thermal stability of NSPR in an N_2_ atmosphere was investigated using TG/DTG analysis to determine the carbonization temperature of the precursor. A continuous weight loss occurred during the thermogravimetric analysis of NSPR, as depicted in Figure 1C. The sample exhibits a rapid weight loss peak (at approximately 200°C) on the DTG curve, which is attributed to the dehydration of the NSPR. Notably, the most of weight loss occurs in the 380°C temperature range, which may be attributed to the volatilization of the light component (hydrocarbons, CO_2_). Above 800°C, there is almost no mass loss, so the carbonization temperature sets at 800°C (Zhang W. et al., 2021).

The mechanism of KOH activation involves multiple simultaneous reactions. The NSPR disintegrates into carbon, volatiles, and gases as the temperature rises in the nitrogen atmosphere, and KOH particles melt into liquid (Chang et al., 2021). KOH and carbon start to react simultaneously (Eq. 6), and this reaction involves numerous reactions between the various intermediates as well as the reduction of the potassium compound to metallic potassium and the oxidation of C to CO_2_ and carbonate. The carbon framework is etched to form a porous structure by the various potassium compounds during KOH activation. Furthermore, the carbon matrix expansion is due to the intercalation of metallic K into the carbon lattice of the carbon matrix (Liu et al., 2020). The overall mechanism of the activation reaction is as follows:

$$2C+6KOH\;\to \;2K_{2}{CO}_{3}+2K+3H_{2}$$ (6)

$$K_{2}{CO}_{3}\;\to \;K_{2}O+{CO}_{2}$$ (7)

$${CO}_{2}+C\;\to \;2CO$$ (8)

$$K_{2}{CO}_{3}+2C\;\to \;2K+3CO$$ (9)

$$C+K_{2}O\;\to \;2K+CO$$ (10)

As seen in Supplementary Figures S1,S2 and Supplementary Table S1, N and S elements in semi-coking wastewater were detected by XRD and GC-MC analysis. Moreover, elemental analysis was employed to investigate the element species and content of the intermediate product NSPR. Supplementary Table S2 illustrates the results, which confirm that the N and S groups in the semi-coking wastewater are successfully incorporated into the NSPR. Simultaneously, XPS analysis revealed that the N and S species surface contents of NSPR are 2.94 and 0.56%, respectively (Table 2), indicating successful *in situ* N and S doping, which is consistent with the results of elemental analysis.

### Morphological and structural characterizations

**FIGURE 2:**
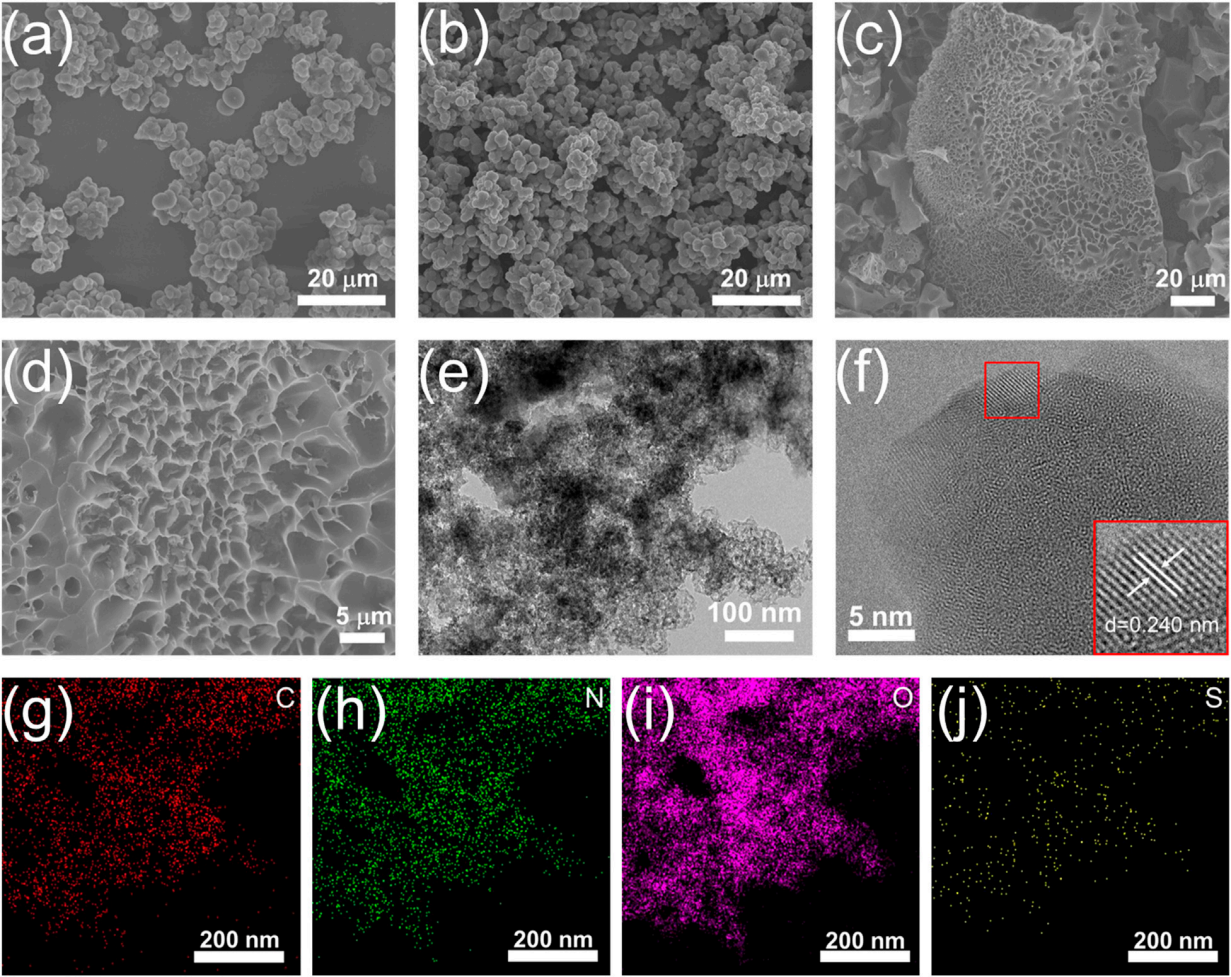
SEM images of **(A)** NSPR and **(B)** NSC **(C,D)** SEM images, **(E)** TEM images, **(F)** HRTEM image, and **(G–J)** EDX elemental mapping images of NSPC.

Figure 2A demonstrates that the NSPR retained its spherical structure even after pyrolysis at 800°C, with relatively smooth surfaces devoid of pores (Figure 2B). Furthermore, no pores are observable in the TEM images (Supplementary Figure S3), demonstrating the crucial role of KOH in the formation of porous structure and high SSA. After water washing, the obtained NSPC displayed interconnected extended layers forming a porous structure (Figure 2C), which was entirely different from the spherical structure of the NSC without KOH activation. The high-resolution SEM image (Figure 2D) and TEM image (Figure 2E) indicate that the honeycomb structure of NSPC is composed of stacked carbon nanosheets, confirming the role of KOH as a template. The HRTEM image depicts that NSPC primarily consists of amorphous porous carbon, and Figure 2F illustrates that the carbon interlayer distance of NSPC is approximately 0.240 nm. This may be because during the KOH activation process, the metal potassium produced at high temperature can penetrate the internal structure of the carbon lattice and distort the carbon layers, resulting in a carbon layer spacing smaller than that of conventional graphite (0.34 nm) (Hunsom and Autthanit, 2013). The EDX elemental mapping images of NSPC (Figures 2G–J) exhibit that a significant amount of N and S atoms are observed and well dispersed in NSPC, demonstrating the doping effect of N and S groups in semi-coking wastewater.

**FIGURE 3:**
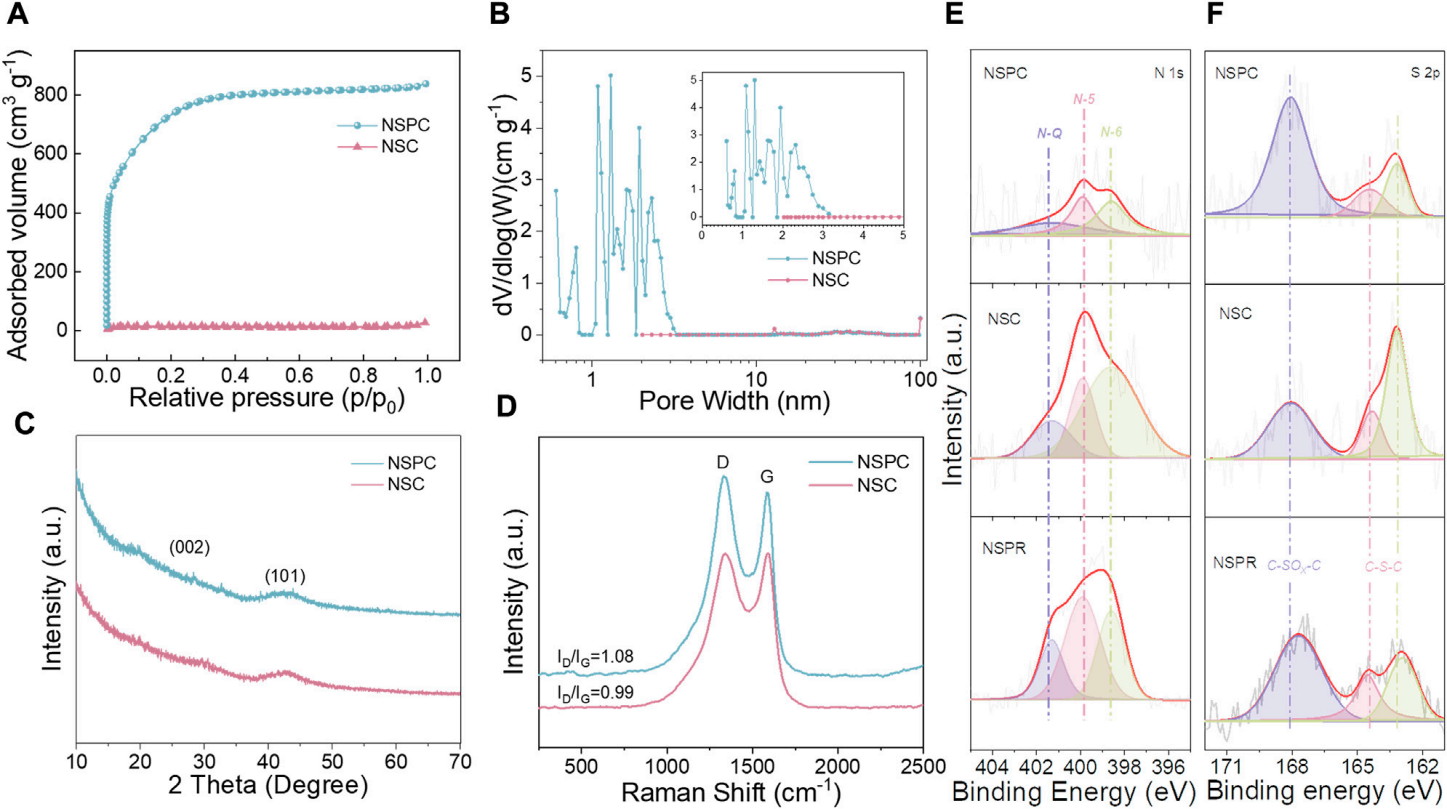
**(A)** N_2_ adsorption-desorption isotherms, **(B)** pore size distribution plots, **(C)** XRD patterns, **(D)** Raman spectra of NSPR and NSC, **(E)** N 1s spectra, **(F)** S 2p spectra of NSPR, NSC, and NSPC.

The SSA and pore size distribution of NSC and NSPC were determined using BET and NLDFT theories, respectively. Table 1 lists the pore parameters of the samples, and Figure 3A demonstrates that NSPC possessed a typical I isotherm. The SSA (2,523 m^2^ g^−1^) and pore volume (1.30 cm^3^ g^−1^) of NSPC are larger than those of NSC (48 m^2^ g^−1^ and 0.04 cm^3^ g^−1^) because of the activator role of KOH. The pore size distribution plots further demonstrate the abundance of micropores and mesopores in NSPC (Figure 3B). The micropores and mesopores of NSPC contribute to its electrochemical performance, with the micropores providing charge storage sites and the mesopores offering electrolyte ion diffusion channels. In contrast, NSC has almost no pore structure without the assistance of KOH. XRD and Raman were employed to characterize the crystal phase structures of the carbons. The XRD patterns of NSC and NSPC (Figure 3C) exhibit two peaks around 24° and 44°, corresponding to the (002) and (100) planes of porous carbon, respectively, representing the amorphous structures of carbon material (Huang et al., 2021). In the Raman spectra (Figure 3D), the ID/IG values of NSC and NSPC are 0.99 and 1.08, respectively, indicating rich defects within the structure of NSPC.

**TABLE 1:** Pore structure parameters of NSC and NSPC.

Sample	S_BET_ ^a^ (m^2^ g^−1^)	V_t_ ^b^ (cm^3^ g^−1^)	Specific surface area^c^ (m^2^ g^−1^)	
			Micropore	Mesopore
NSC	48	0.04	-	48
NSPC	2,523	1.30	1875	648

a Total specific surface area, calculated by Brunauer-Emmett-Teller (BET) method.

b Total pore volume, determined at a relative pressure of 0.99;

c The pore volume of micropore and meso-/macropore, calculated by t-plot method.

Semi-coking wastewater, produced by the carbonization of coal at low to medium temperatures, contains naturally occurring N and S elements (Bokun et al., 2020). The chemical states of elements were also investigated using XPS. Supplementary Figure S4 demonstrates the successful N and S self-doping by indicating the existence of N and S species in the three samples. The NSC and NSPC possess 2.19 and 0.17% of N contents, respectively, with 0.36 and 0.32% of S contents (Table 2). The NSPC exhibits less N and S content after activation with potassium hydroxide than the NSC, implying that the KOH activator could reduce the N and S heteroatoms contents. During the carbonization process, the pores generated by the activation of KOH resulted in the exposure of N and S atoms on the surface. The exposed N and S atoms are further annealed and thus decomposed so KOH activator could reduce the N and S heteroatoms contents. The N 1*s* spectra can be deconvoluted into three peaks (Figure 3E), indicating pyridinic-N (N-6), pyrrolic-N (N-5), and quaternary-N (N-Q) (Zhang et al., 2022). NSPC exhibits less N-Q content than NSC, illustrating that the carbon skeletons possess more defects and disordered structures due to the activation role of KOH. N-6 and N-5 can contribute pseudo-capacitance, while N-Q can facilitate electron transfer across the electrode-electrolyte interface, enhancing the materials’ electrical conductivity.

**TABLE 2:** Surface element composition determined by XPS and relative contents of functional groups in N 1s and S 2p peaks of samples.

Sample	C	N	O	S	N-6	N-5	N-Q	C-SO_X_-C	S 2p_1/2_	S 2p_2/3_
	at. (%)	at. (%)	at. (%)	at. (%)	at. (%)	at. (%)	at. (%)	at. (%)	at. (%)	at. (%)
NSPR	77.07	2.94	19.43	0.56	0.64	1.37	0.93	0.31	0.11	0.14
NSC	86.94	2.19	10.51	0.36	0.37	0.52	1.30	0.13	0.05	0.18
NSPC	90.44	0.71	8.53	0.32	0.26	0.21	0.24	0.23	0.04	0.05

In the XPS spectrum of S 2*p* (Figure 3F), a peak at 168.07 eV is attributed to C-SO_x_-C, and the other two peaks at 164.3 and 163.2 eV are assigned to S 2*p*_1/2_ and S 2*p*_3/2_, respectively (Liu et al., 2022). It demonstrates that when the NSPC is activated with KOH, more S heteroatoms are doped into it, leading to the formation of C-SO_x_-C. The result confirms the successful doping of S onto NSPR. It is consistent with the Elemental analysis and SEM results.

### Electrochemical performance of the three-electrode system

**FIGURE 4:**
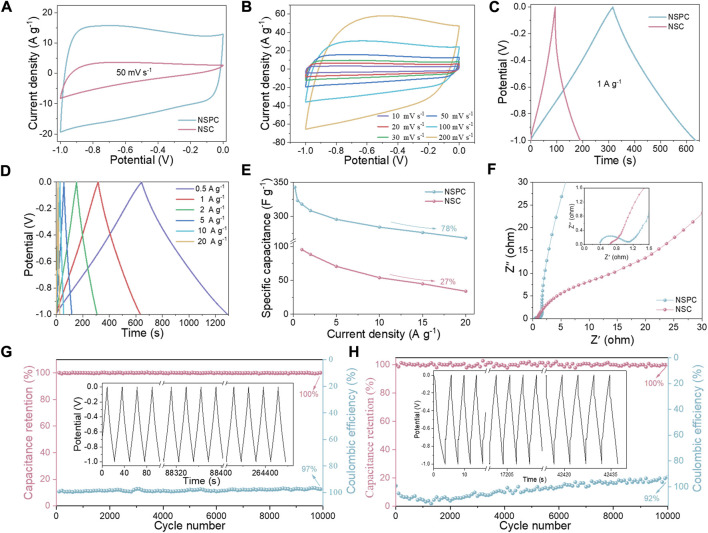
Comparative electrochemical performance of NSC and NSPC in 6 M KOH electrolyte: **(A)** CV curves at 50 mV s^−1^, **(B)** CV curves of NSPC at scan rates ranging from 10 to 200 mV s^−1^, **(C)** GCD profile at 1 A g^−1^, **(D)** GCD curves of NSPC at the current density ranging of 0.5–20 A g^−1^, **(E)** Specific capacitances at different current densities, **(F)** Nyquist plots, and **(G–H)** cycling stability and Coulombic efficiency of NSPC and NSC at 20 A g^−1^ (inset are GCD curves after 10,000 cycles).

The electrochemical performance of the three-electrode system was first investigated. The capacitance is dominated by double layer capacitance and also contains pseudo-capacitance generated by N-S heteroatoms doping, as illustrated in Figure 4A, which depicts the NSC and NSPC in a quasi-rectangular shape at a scan rate of 50 mV s^−1^. Furthermore, when compared to NSC, the NSPC has a larger encircled area, indicating a higher capacitance. The CV curves of NSPC at different scan rates can exhibit a great rectangular shape in Figure 4B, illustrating its remarkable rate ability. The GCD curves of NSC and NSPC display the isosceles triangle shapes related to double layer capacitance in Figure 4C. The GCD curves of NSPC (Figure 4D) reveal no apparent IR drop, implying that NSPC possesses small internal resistance. Figure 4E demonstrates the remarkable rate capability (78%) of NSPC, which reduces the specific capacitance from 343 F g^−1^ at 0.2 A g^−1^–268 F g^−1^ at 20 A g^−1^. However, the specific capacitance of NSC drops sharply from 117.2 F g^−1^ at 0.2 A g^−1^–20 F g^−1^ at 20 A g^−1^, displaying a low-rate capability of only 27%. High-rate capability is one of the most significant factors for capacitors in practical applications.

The electrolyte transport mechanism was investigated using EIS analysis to better comprehend the electrolyte ion transport properties. As illustrated in Figure 4F, NSPC exhibits an approximate semicircle, whereas NSC reveals almost no semicircle in the high-frequency range, and NSPC shows an almost vertical line, while NSC demonstrates a line with a slope of less than 45^o^ in the low-frequency range. The assembled equivalent electric circuit is depicted in Supplementary Figure S5 to quantify the resistance, and the resulting parameters are given in Supplementary Table S3. The real axis intercept in the high-frequency region represents solution resistance (R_s_) (Wei et al., 2019). The diameter of the semicircle represents the charge transfer resistance (R_ct_) at the electrode/electrolyte interface (Sun et al., 2022). NSPC shows a low R_s_ (0.4 Ω) and a minor R_ct_ (0.7 Ω), indicating a low resistance owing to the fast ion transfer in the porous structure. Moreover, the straight lines highlight electrode material with rapid charge transfer kinetics. Conversely, NSC displays higher R_ct_ values than NSPC (0.8 Ω). NSC has less porous surfaces that impede the easy entry of electrolyte ions, leading to higher resistance. The NSPC electrode retained 100% capacitance even after 10,000 continuous GCD cycles with a Coulombic efficiency of 97% at 20 A g^−1^, as shown in Figure 4G. This long cycling stability may be attributable to its abundant SSA-ordered porosity. Supplementary Figure S6 displays the CV and GCD profiles of NSC. In comparison to NSPC, the CV curves of NSC deviate more at higher scan rates, implying the low-rate capability of the materials. Meanwhile, Figure 4H demonstrates that the NSC has poor stability, with a Coulombic efficiency of 92% after 10,000 cycles.

### Electrochemical performance of NSPC symmetric supercapacitor

**FIGURE 5:**
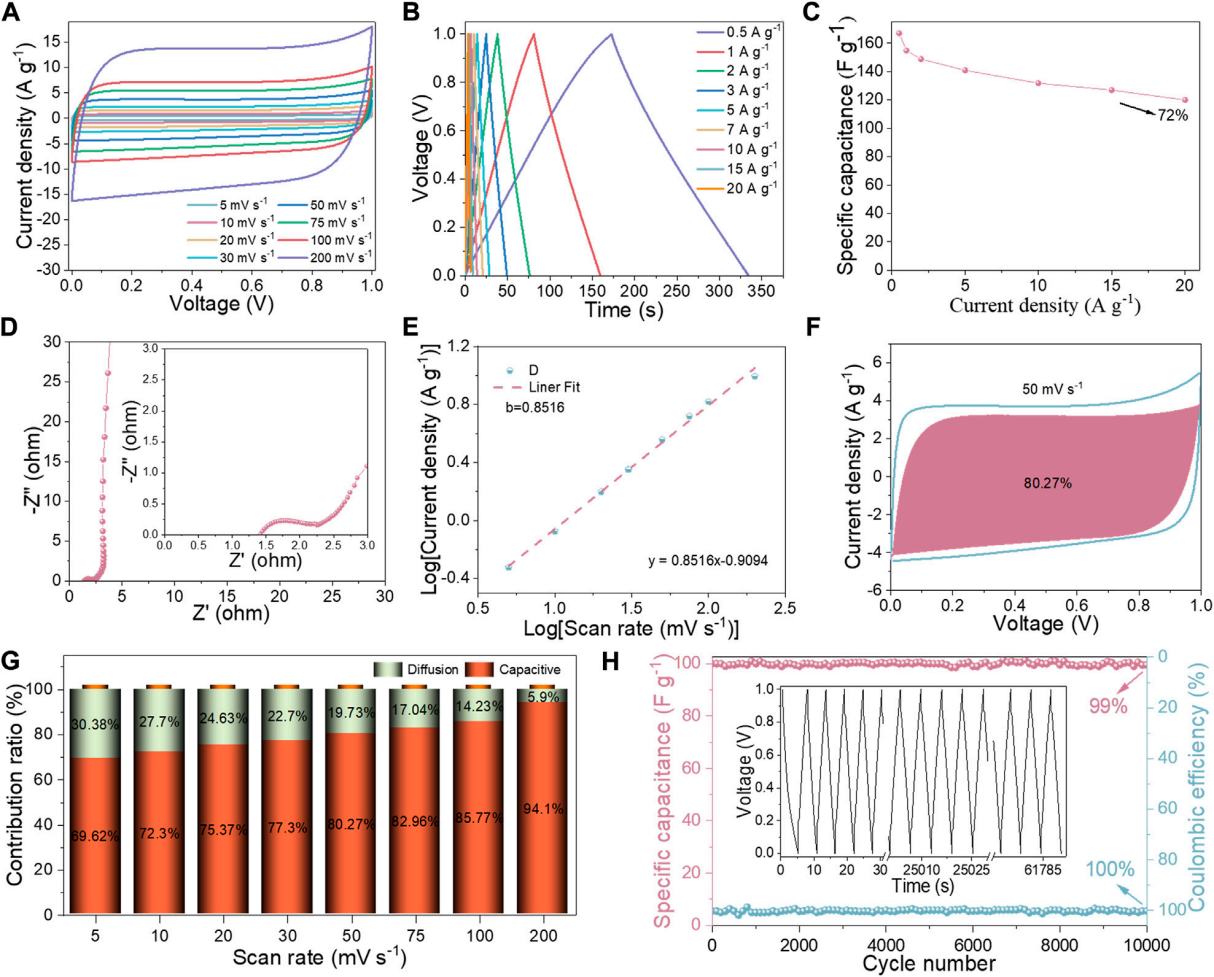
Electrochemical performance of the NSPC symmetric device in 6 M KOH electrolyte: **(A)** CV curves at different sweep rates, **(B)** GCD curves at different current densities, **(C)** NSPC Specific capacitances at different current densities, **(D)** Nyquist plots, **(E)** linear plot of log *i* vs log *v* in charge processes, **(F)** capacitive contribution at 50 mV s^−1^, **(G)** diffusion- and capacitive-controlled contribution ratio at different scan rates, and **(H)** cycling stability and Coulombic efficiency of NSPC at 20 A g^−1^ (inset is GCD curves after 10,000 cycles).

The obtained NSPC is thought to be outstanding electrode material owing to its high SSA (2,523 m^2^ g^−1^), enormous V_t_ (1.30 cm^3^ g^−1^), and reasonable N, S doping. The fabricated NSPC symmetric supercapacitor displays outstanding electrochemical performance and can be operated between 0 and 1 V. The CV curves at different scan rates are illustrated in Figure 5A; the NSPC symmetric supercapacitor could maintain its rectangle shape even at 200 mV s^−1^, indicating double-layer capacitance behaviors and excellent rate performance (Zhang J. et al., 2021). The tiny distortion may be relevant to the pseudo-capacitance caused by N-S self-doping. The GCD curves shown in Figure 5B again validated the outstanding rate performance of the NSPC symmetric supercapacitor. Figure 5C depicts the calculated specific capacitances of the NSPC symmetric supercapacitor from the GCD curves. The specific capacitance of the NSPC symmetric supercapacitor reaches 167 F g^−1^ at 0.5 A g^−1^ and can still reach 120 F g^−1^ at 20 A g^−1^, demonstrating superior capacitance retention (72%). The EIS analysis is employed to better understand the rate capacitance of the NSPC symmetric supercapacitor. The R_s_ and R_ct_ were 1.4 and 0.8 Ω, respectively, as depicted in Figure 5D, implying high conductivity and efficient charge transfer owing to its suitable pore structures.

The NSPC symmetric supercapacitor exhibits remarkable double-layer capacitance as well as clear pseudo-capacitance originating from N, S heteroatom doping, as demonstrated by the above analysis. Thereby, the charging kinetics of the NSPC symmetric supercapacitor are assessed qualitatively using Eqs 11, 12 (Tiwari et al., 2021):

$$i=av^{b}$$ (11)

log(i)=log⁡a+blog⁡v (12)

where *i* and *v* represent the current peak value and scan rate, respectively, *a* and *b* are calculated by Equation 12. Typically, when *b* is are close to 0.5, the capacitor behavior is dominated by pseudo-capacitive. When *b* approaches 1, double-layer capacitance controls the capacitor’s behavior. The calculated b values of the NSPC symmetric supercapacitor are 0.8516 in Figure 5E, signifying that its kinetic behavior is based on double-layer capacitance. In addition, the capacitance contribution from double-layer capacitance and pseudo-capacitance of NSPC-SS is utilized to conduct a more precise quantitative analysis by Dunn’s method, as illustrated in Eqs 13, 14 (Wang et al., 2007):

$$i=k_{1}v+k_{2}v^{1/2}$$ (13)

$$i/v^{1/2}=k_{1}v^{1/2}+k_{2}$$ (14)

where *k*_*1*_ *v* and *k*_*2*_ *v* ^*1/2*^ represent the double-layer capacitance contribution and pseudo-capacitance contributions, respectively. Figure 5F represents the current contribution of the NSPC symmetric supercapacitor at 50 mV s^−1^, revealing that the current contribution is driven by the double-layer capacitance. The results of calculations at different scan rates are depicted in Figure 5G; its double-layer capacitance improves to over 94.1% at a scan rate of 200 mV s^−1^, corresponding to its high rate capability. Furthermore, the symmetric NSPC supercapacitor exhibits outstanding cycling stability and reversible performance (Figure 5H). The NSPC symmetric supercapacitor has retained a Coulombic efficiency of 100% and capacitance retention of 99% after 10,000 charge/discharge cycles at 20 A g^−1^. Both GCD plots maintain a triangular shape (the insert in Figure 5H), confirming their superior electrochemical stability.

**FIGURE 6:**
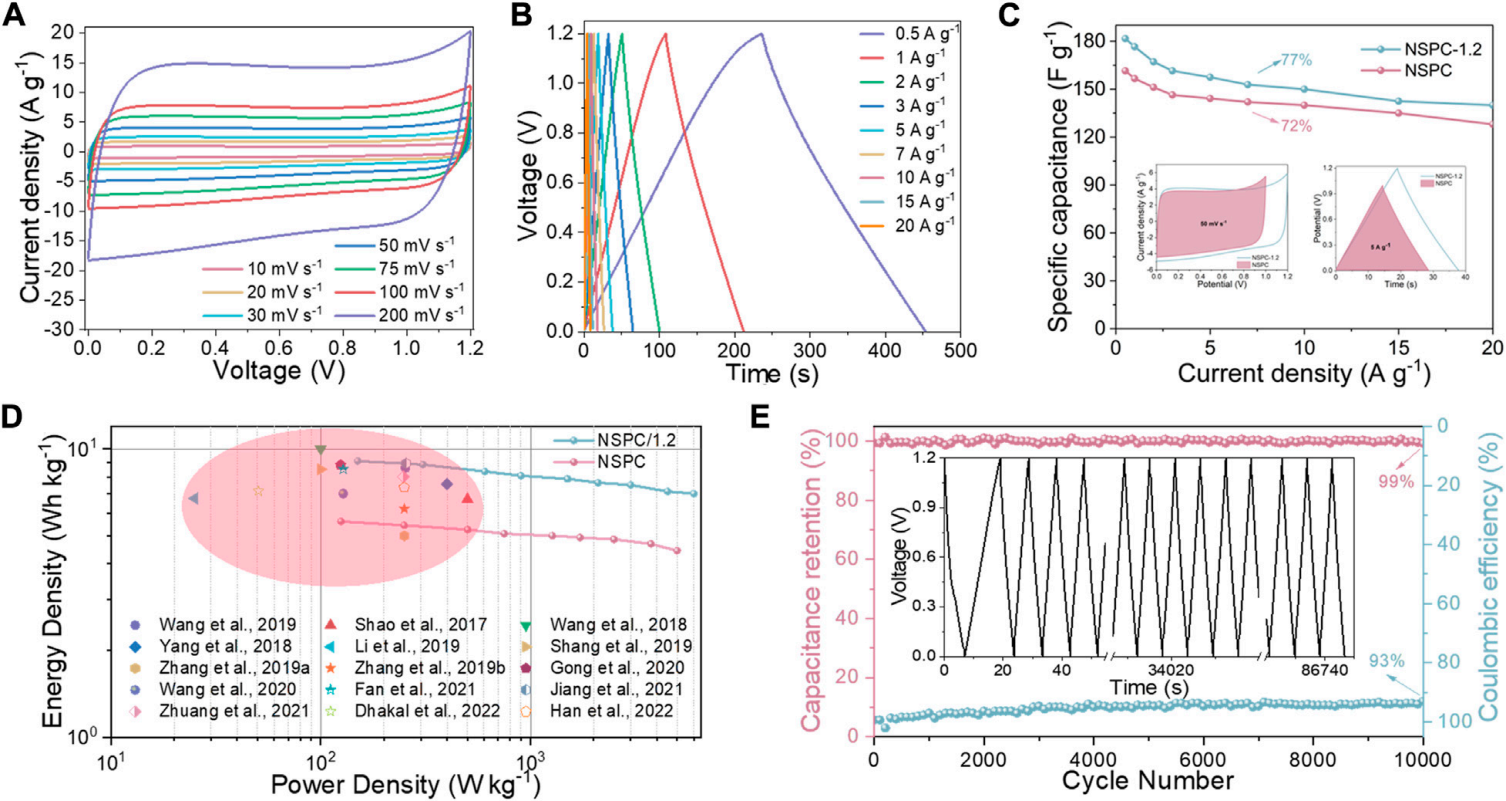
Performance of the NSPC symmetric device at 1.2 V in 6 M KOH electrolyte: **(A)** CV curves at different scan rates, **(B)** GCD curves at different current densities, **(C)** Rate capability at 1 and 1.2 V (insets are CV curves at 50 mV s^−1^ and GCD curves at 5 A g^−1^), **(D)** Ragone plots in comparison with other works, and **(E)** cycling performance and Coulombic efficiency at 20 A g^−1^ (inset is GCD curves after 10,000 cycles).

Considering that increasing the working voltage of the capacitor can increase its energy density. Therefore, the electrochemical performance of the NSPC/1.2 symmetric supercapacitor is investigated at an operating voltage of 1.2 V. The NSPC/1.2 symmetric supercapacitor exhibits an approximate quasi-rectangular shape from 5 to 200 mV s^−1^, as depicted in Figure 6A, revealing its excellent rate performance. The GCD curves of the NSPC/1.2 symmetric supercapacitor have nearly symmetrical triangles (Figure 6B), confirming its outstanding reversibility. The specific capacitance of NSPC/1.2 is determined to be 182 F g^−1^ at 0.5 A g^−1^ and 140 F g^−1^ at 20 A g^−1^. The capacitance retention could reach 77% at 20 A g^−1^ (Figure 6C), indicating a remarkable rate capability. Additionally, after 10,000 cycles at 20 A g^−1^, the NSPC/1.2 symmetric supercapacitor retained 99% capacitance and 93% Coulombic efficiency (Figure 6E). Although the Coulombic efficiency is lower than that obtained at 1 V operating voltage, the electrochemical stability of the NSPC/1.2 symmetric supercapacitor is still remarkable. All of these results indicate that at an operating voltage of 1.2 V, heteroatoms as electroactive sites do not undergo significant water splitting.

The relationship between the energy and power densities of NSPC/1.2 symmetric supercapacitors was investigated. The Ragone diagrams of the NSPC and NSPC/1.2 symmetric supercapacitors are depicted in Figure 6D, and the NSPC/1.2 symmetric supercapacitor has an energy density as high as 9.1 Wh kg^−1^ at a power density of 150 W kg^−1^. Furthermore, even at a high-power density (6.0 kW kg^−1^), the energy density remains at 7.0 Wh kg^−1^. This high energy density makes NSPC/1.2 symmetric supercapacitor comparable to other carbon-based materials for supercapacitors in alkaline electrolytes (Shao et al., 2017; Wang et al., 2018; Yang et al., 2018; Zhang D. et al., 2019; Zhang X. et al., 2019; Li et al., 2019; Shang et al., 2019; Gong et al., 2020; Wang et al., 2020; Wang et al., 2020; Fan et al., 2021; Jiang et al., 2021; Zhuang et al., 2021; Dhakal et al., 2022; Han et al., 2022), and the main performance parameters are compared in Supplementary Table S4. These results confirm the feasibility of phenolic wastewater-derived carbon materials for energy storage applications.

## Conclusion

In summary, the N, S self-doped porous carbon was first prepared by formaldehyde pretreatment-assisted KOH activation from semi-coking wastewater. The as-prepared NSPC exhibited appropriate heteroatom doping, a large SSA, and an optimal pore size distribution, which improved electrical conductivity and reversibility. The NSPC electrode had a higher specific capacitance of 323 F g^−1^ at 0.5 A g^−1^. Furthermore, the NSPC symmetric supercapacitor demonstrated an energy density of 9.1 Wh kg^−1^ at an operating voltage of 1.2 V and a power density of 0.15 kW kg^−1^. The capacitance retention remained at 99% after 10,000 charge/discharge cycles at 20 A g^−1^. This work not only provided a facile and cost-effective route to large-scale treatment of semi-coking wastewater, but also a sustainable, simple, and viable manufacturing process, semi-coking wastewater-derived NSPC would be a cost-effective and promising candidate for capacitor electrode material.

## Data Availability

The original contributions presented in the study are included in the article/Supplementary Material, further inquiries can be directed to the corresponding author.
