# Supplementary material for: Self-doped N, S porous carbon from semi-coking wastewater-based phenolic resin for supercapacitor electrodes
Source: Front Chem. 2022 Oct 6;10:1021394. doi: 10.3389/fchem.2022.1021394 (PMC9583164; doi:10.3389/fchem.2022.1021394)
Supplement: Supplementary file 1 [file DataSheet1.docx]

**Self-doped N, S porous carbon from semi-coking wastewater-based phenolic resin for supercapacitor electrodes**

Long Yan ^a,^ ^[[1]](#footnote-1)^, Xianjie Wang ^a,^ ^†^, Yufei Wang ^a, b^ ^[[2]](#footnote-2)^*, Jian Li ^a, b^, Qianqian Liu ^a^, Xiang Zhong ^a^, Yuan Chang ^a^, Qingchao Li ^a^, Santosh Kumar Verma ^a^

*^a^ Shaanxi Key Laboratory of Low Metamorphic Coal Clean Utilization, School of Chemistry and Chemical Engineering,* *Yulin University, Yulin 719000, PR China*

*^b^ National Engineering Research Center of Coal Preparation and Purification, China University of Mining and Technology, Xuzhou 221116, PR China*

^*^ Corresponding author. E-mail: wangyufei0003@163.com (Y. Wang)

**Supporting information**

**Figure and Table caption**

**Fig. S1.** XRD pattern of semi-coking wastewater.

**Fig. S2.** Total ion chromatograms of semi-coking wastewater

**Fig. S3.** TEM image of NSC

**Fig. S4.** XPS survey spectra of NSPR, NSC and NSPC.

**Fig. S5.** The equivalent circuit model.

**Fig. S6.** Electrochemical performance of NSC in a 6 M KOH electrolyte in the three-electrode setup: (a) CV curves, (b) GCD profiles.

**Table S1.** The main organic compounds and their relative contents in the semi-coking wastewater.

**Table S2.** C, N, O, and H contents evaluated from elemental analysis.

**Table S3.** The calculated parameters of the equivalent circuit for the obtained samples.

**Table S4.** Comparison of the electrochemical performances for carbon-based materials assembled into supercapacitors in 6M KOH electrolyte.

**Fig. S1** XRD pattern of semi-coking wastewater.

Use a capillary to measure a small amount of semi-coking wastewater on the zero background sample stage, and repeat 2 to 3 times to enrich the sample. After the liquid had evaporated, the crystals remaining on the sample stage were detected by XRD.


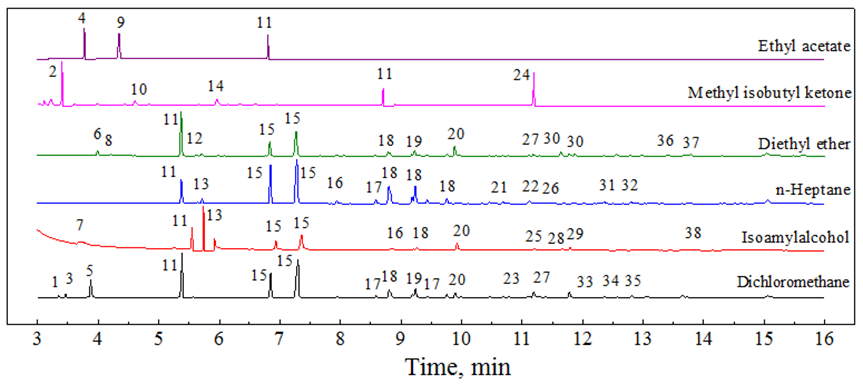


**Fig. S2** Total ion chromatograms of semi-coking wastewater.

Using dichloromethane, isoamylol, n-heptane, diethyl ether, MIBK and ethyl acetate as the organic solvents, the organic pollutants in semi-coking wastewater are extracted and analyzed by GC-MS.


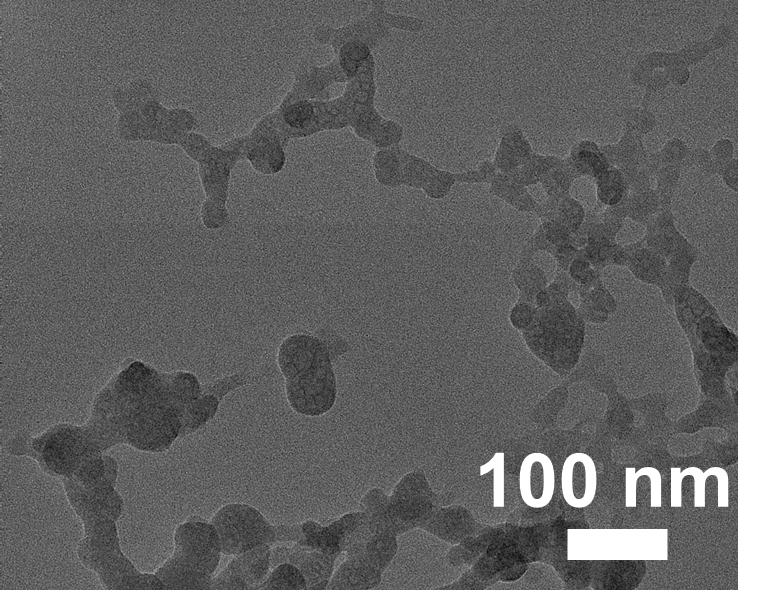


**Fig. S3** TEM image of NSC.

**Fig. S4** XPS survey spectra of NSPR, NSC and NSPC.


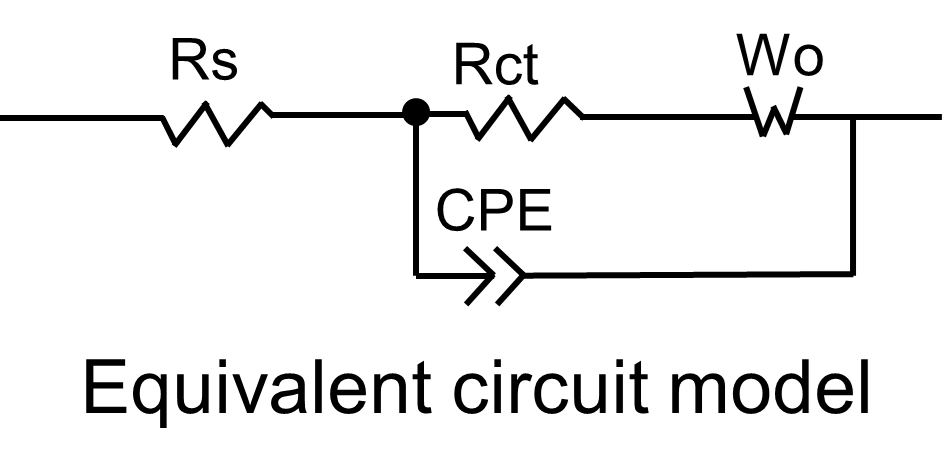


**Fig. S5** The equivalent circuit model

**Fig. S6** Electrochemical performance of NSC in a 6 M KOH electrolyte in the three-electrode setup: (a) CV curves, (b) GCD profiles.

**Table S1** The main organic compounds and their relative contents in the semi-coking wastewater.

| No. | Compounds | Retention time/min | Cdichloromethane/% | Cisoamylol/% | Cn-heptane/% | Cdiethyl ether/% | CMIBK/% | Cethyl acetate/% |
| --- | --- | --- | --- | --- | --- | --- | --- | --- |
| 1 | ethylbenzene | 3.352 | 0.82 |  |  |  |  |  |
| 2 | 3,5-dimethyl-2-cycloethene-1-methyloxime | 3.408 |  |  |  |  | 12.03 |  |
| 3 | 1,3-dimethyl benzene | 3.464 | 1.45 |  |  |  |  |  |
|  |  | 3.833 | 0.59 |  |  |  |  |  |
| 4 | 3-ethoxy-4-methyl phenol | 3.774 |  |  |  |  |  | 4.78 |
| 5 | cyclohexanone | 3.878 | 8.28 |  |  |  |  |  |
| 6 | ethyl acetate | 3.989 |  |  |  | 2.86 |  |  |
| 7 | isopentanol | 3.703 |  | 5.10 |  |  |  |  |
|  |  | 3.756 |  | 3.94 |  |  |  |  |
| 8 | 2-aminoisopropyl ether | 4.208 |  |  |  | 0.98 |  |  |
| 9 | p-benzenediol | 4.346 |  |  |  |  |  | 7.89 |
| 10 | 4-methyl phenol | 4.611 |  |  |  |  | 5.56 |  |
| 11 | phenol | 5.373 | 17.58 |  | 9.14 | 27.62 | 12.73 | 14.56 |
|  |  | 5.558 |  | 9.73 |  |  |  |  |
| 12 | pentyl 2-hydroxypropanoate | 5.708 |  |  |  | 1.31 |  |  |
| 13 | isopentyl ether | 5.742 | 11.64 | 1.87 |  |  |  |  |
|  |  | 5.926 | 10.48 |  |  |  |  |  |
| 14 | 5-hydroxy-2-pentanone | 5.963 |  |  |  |  | 7.35 |  |
| 15 | 3-methyl phenol | 6.835 | 12.11 |  | 17.35 | 9.71 |  |  |
|  |  | 6.935 |  | 9.56 |  |  |  |  |
|  |  | 7.273 | 29.76 |  | 28.06 | 24.23 |  |  |
|  |  | 7.362 |  | 23.02 |  |  |  |  |
| 16 | 2,6-dimethyl phenol | 7.947 |  |  | 1.42 |  |  |  |
|  |  | 8.836 |  | 4.58 |  |  |  |  |
| 17 | 2-ethyl phenol | 8.587 | 0.88 |  | 1.89 |  |  |  |
|  |  | 9.241 | 4.82 |  |  |  |  |  |
|  |  | 9.442 | 0.87 |  |  |  |  |  |
|  |  | 9.760 | 1.45 |  |  |  |  |  |
| 18 | 2,3-dimethyl phenol | 8.794 | 5.95 |  | 12.85 | 4.52 |  |  |
|  |  | 9.261 |  | 3.20 |  |  |  |  |
|  |  | 9.228 | 5.95 |  | 8.86 | 3.383 |  |  |
|  |  | 9.438 |  |  | 1.82 |  |  |  |
|  |  | 9.754 |  |  | 2.55 | 1.37 |  |  |
| 19 | 3-ethyl phenol | 9.183 | 1.96 |  | 3.11 | 1.31 |  |  |
| 20 | o-benzenediol | 9.889 | 2.85 |  |  | 6.91 |  |  |
|  |  | 9.929 |  | 8.14 |  | 6.91 |  |  |
| 21 | oxalic acid-2-isopropylphenylpentyl ester | 10.469 |  |  | 0.80 |  |  |  |
|  |  | 10.690 |  |  | 1.03 |  |  |  |
|  |  | 10.780 |  |  | 0.63 |  |  |  |
| 22 | 1,5,5-trimethyl-6-methylene-1-cyclohexene | 11.127 |  |  | 1.84 |  |  |  |
| 23 | 3-methanol phenol | 11.194 | 3.02 |  |  |  |  |  |
| 24 | o-methyl phenol |  |  |  |  |  | 23.55 |  |
| 25 | 2-ethoxy-4-methyl phenol | 11.208 |  | 2.41 |  |  |  |  |
| 26 | 4-heptyloxy-1-ethanalylenzene | 11.300 |  |  | 1.00 |  |  |  |
| 27 | 4-methylo-benzenediol | 11.187 |  |  |  | 1.09 |  |  |
|  |  | 11.774 | 3.27 |  |  | 1.84 |  |  |
| 28 | p-phenol | 11.640 |  | 2.46 |  | 3.35 |  |  |
| 29 | 3-ethoxy-5-hydroxymethyl phenol | 11.795 |  | 3.52 |  |  |  |  |
| 30 | 2-methyl-1,3-benzenediol | 11.874 |  |  |  | 1.91 |  |  |
|  |  | 12.565 | 3.02 |  |  | 1.22 |  |  |
| 31 | p-propene phenol | 12.366 |  |  | 0.78 |  |  |  |
| 32 | 1-hydroxyhydrindene | 12.802 |  |  | 1.07 |  |  |  |
| 33 | 3-propene phenol | 12.817 | 1.10 |  |  |  |  |  |
| 34 | 2,6-dimethyl-1,4-benzenediol | 13.046 | 0.82 |  |  |  |  |  |
| 35 | 4-ethyl-1,2-benzenediol | 13.649 | 1.40 |  |  |  |  |  |
| 36 | 3-hydroxy-D-tyrosine | 13.707 |  |  |  | 0.83 |  |  |
| 37 | 9-octadecene-2-phenyl-1,3-dioxygenolanemethyl ester | 13.784 |  |  |  | 1.16 |  |  |
| 38 | butanoate-4-octyl ester | 13.858 |  | 0.97 |  |  |  |  |

**Table S2** C, N, O, and H contents evaluated from elemental analysis.

| Sample | C（wt.%） | N（wt.%） | H（wt.%） | S（wt.%） | O（wt.%） |
| --- | --- | --- | --- | --- | --- |
| NSPR | 63.3355 | 3.39 | 5.716 | 1.642 | 27.787 |
| NSC | 77.143 | 2.139 | 1.713 | 1.229 | 14.181 |
| NSPC | 86.505 | 0.76 | 0.509 | 0.914 | 1.913 |

**Table S3** The calculated parameters of the equivalent circuit for the obtained samples.

| **Sample** | R_s_ (Ω) | R_ct_ (Ω) |
| --- | --- | --- |
| NSC | 0.6 | 0.8 |
| NSPC | 0.4 | 0.7 |

**Table S4** Comparison of the electrochemical performances for carbon-based materials assembled into supercapacitors in 6M KOH electrolyte.

| Raw Materials | Surface area  (m^2^ g^-1^) | Electrolyte | Capacitance  (F g^-1^) | E *^c^*  (Wh kg^-1^) | P *^c^*  (W kg^-1^) | Reference |
| --- | --- | --- | --- | --- | --- | --- |
| semi-coking wastewater | 2523 | KOH | 323 *^a^*  (0.5 A g^-1^) | 9.1 | 150 | In this work |
| glucose | 1515.6 | KOH | 313 *^a^*  (0.1 A g^-1^) | 8.6 | 253 | Wang et al., 2019 |
| lotus calyx biowaste | 798 | KOH | 223 *^a^*  (1 A g^-1^) | 6.7 | 500 | Shao et al., 2017 |
| chitosan | 1770 | KOH | 332 *^a^*  (1 A g^-1^) | 10 | 100 | Wang et al., 2018 |
| Coal tar pitch | 2059 | KOH | 313 *^a^*  (1 A g^-1^) | 7.54 | 400 | Yang et al., 2018 |
| phenolic-resin-based analogues | 1462 | KOH | 194 *^a^*  (0.1 A g^-1^) | 6.74 | 25 | Li et al., 2019 |
| Glucose | 940 | KOH | 367 *^b^*  (0.3 A g^-1^) | 8.5 | 100 | Shang et al., 2019 |
| carbon microspheres | 679 | KOH | 279.4 *^a^*  (0.5 A g^-1^) | 5 | 250 | Zhang et al., 2019a |
| PMMA, resorcinol and formaldehyde | 670 | KOH | 157.9 *^b^*  (1 A g^-1^) | 6.2 | 250 | Zhang et al., 2019b |
| anthracite | 1581 | KOH | 470.7 *^a^*  (0.5 A g^-1^) | 8.8 | 124.4 | Gong et al., 2020 |
| polymer/graphene oxide hydrogels | 1009 | KOH | 203*^b^*  (0.5 A g^-1^) | 7 | 128 | Wang et al., 2020 |
| Coal tar pitch | 2254 | KOH | 244 *^b^*  (0.5 A g^-1^) | 8.5 | 128 | Fan et al., 2021 |
| Coal tar pitch | 3343 | KOH | 308 *^a^*  (1 A g^-1^) | 8.92 | 254.9 | Fan et al., 2021 |
| Asphaltene | 2233 | KOH | 301 *^a^*  (1 A g^-1^) | 8 | 248 | Zhuang et al., 2021 |
| Coal tar pitch | 1181 | KOH | 172 *^a^*  (1 A g^-1^) | 7.15 | 50.4 | Dhakal et al., 2022 |
| Cultivated ramie | 1529 | KOH | 212 *^a^*  (0.5 A g^-1^) | 7.36 | 250 | Han et al., 2022 |

*^(a^*^)^ Capacitance with three-electrode system, *^(b)^* Capacitance with two-electrode system, and *^(c)^* Maximum energy density of the optimum sample

**References**

Zhang, X., Li, H., Qin, B., Wang, Q., Xing, X., Yang, D., et al. (2019b). Direct synthesis of porous graphitic carbon sheets grafted on carbon fibers for high-performance supercapacitors. *Journal of Materials Chemistry A* 7**,** 3298-3306. Doi:10.1039/c8ta11844b.

Dhakal, G., Mohapatra, D., Kim, Y.I., Lee, J., Kim, W.K., and Shim, J.J. (2022). High-performance supercapacitors fabricated with activated carbon derived from lotus calyx biowaste. *Renewable Energy* 189, 587-600. Doi:10.1016/j.renene.2022.01.105.

Gong, Y., Li, D., Fu, Q., Zhang, Y., and Pan, C. (2020). Nitrogen Self-Doped Porous Carbon for High-Performance Supercapacitors. *ACS Applied Energy Materials* 3**,** 1585-1592. Doi:10.1021/acsaem.9b02077.

Jiang, Y., He, Z., Du, Y., Wan, J., Liu, Y., and Ma, F. (2021). In-situ ZnO template preparation of coal tar pitch-based porous carbon-sheet microsphere for supercapacitor. *J Colloid Interface Sci* 602**,** 721-731. Doi:10.1016/j.jcis.2021.06.037.

Li, X., Song, Y., You, L., Gao, L., Liu, Y., Chen, W., et al. (2019). Synthesis of Highly Uniform N-Doped Porous Carbon Spheres Derived from Their Phenolic-Resin-Based Analogues for High Performance Supercapacitors. *Industrial & Engineering Chemistry Research* 58**,** 2933-2944. Doi:10.1021/acs.iecr.8b04823.

Yang, W., Yang, W., Kong, L., Song, A., Qin, X., and Shao, G. (2018). Phosphorus-doped 3D hierarchical porous carbon for high-performance supercapacitors: A balanced strategy for pore structure and chemical composition. *Carbon* 127**,** 557-567. Doi:10.1016/j.carbon.2017.11.050

Zhang, D., Wang, J., He, C., Wang, Y., Guan, T., Zhao, J., et al. (2019a). Rational Surface Tailoring Oxygen Functional Groups on Carbon Spheres for Capacitive Mechanistic Study. *ACS Appl Mater Interfaces* 11**,** 13214-13224. Doi:10.1021/acsami.8b22370.

Shang, Y., Hu, X., Li, X., Cai, S., Liang, G., Zhao, J., et al. (2019). A facile synthesis of nitrogen-doped hierarchical porous carbon with hollow sphere structure for high-performance supercapacitors. *Journal of Materials Science* 54**,** 12747-12757. Doi:10.1007/s10853-019-03744-w.

Han, G., Jia, J., Liu, Q., Huang, G., Xing, B., Zhang, C., et al. (2022). Template-activated bifunctional soluble salt ZnCl_2_ assisted synthesis of coal-based hierarchical porous carbon for high-performance supercapacitors. *Carbon* 186**,** 380-390. Doi:10.1016/j.carbon.2021.10.042.

Wang, M., Yang, J., Liu, S., Li, M., Hu, C., and Qiu, J. (2020). Nitrogen-doped hierarchically porous carbon nanosheets derived from polymer/graphene oxide hydrogels for high-performance supercapacitors. *J Colloid Interface Sci* 560**,** 69-76. Doi:10.1016/j.jcis.2019.10.037.

Fan, Y.A., Kll, A., Xywab, C., Zcf, A., Jhl, A., Qqk, A., et al. (2021). Fabrication of N/O self-doped hierarchical porous carbons derived from modified coal tar pitch for high-performance supercapacitors. *Fuel* 310, 122418. Doi:10.1016/j.fuel.2021.122418.

Shao, J., Ma, F., Wu, G., Dai, C., Geng, W., Song, S., et al. (2017). In-situ MgO (CaCO_3_) templating coupled with KOH activation strategy for high yield preparation of various porous carbons as supercapacitor electrode materials. *Chemical Engineering Journal* 321**,** 301-313. Doi:10.1016/j.cej.2017.03.092.

Zhuang, Q.Q., Cao, J.P., Wu, Y., Zhao, X.Y., and Bai, H.C. (2021). Direct synthesis of oxygen-enriched 3D porous carbons via NaCl template derived from oxidized coal tar pitch for excellent cycling stability electric double layer capacitor. *Journal of Power Sources* 508**,** 230330. Doi:10.1016/j.jpowsour.2021.230330.

Wang, D., Wang, Y., Chen, Y., Liu, W., Wang, H., Zhao, P., et al. (2018). Coal tar pitch derived N-doped porous carbon nanosheets by the in-situ formed g-C_3_N_4_ as a template for supercapacitor electrodes. *Electrochimica Acta* 283**,** 132-140. Doi:10.1016/j.electacta.2018.06.151.

Wang, Q., Liu, F., Jin, Z., Qiao, X., Huang, H., Chu, X., et al. (2020). Hierarchically Divacancy Defect Building Dual‐Activated Porous Carbon Fibers for High‐Performance Energy‐Storage Devices. *Advanced Functional Materials*. 30, 2002580. Doi:10.1002/adfm.202002580.

1. These authors contributed equally to this work and share first authorship. [↑](#footnote-ref-1)
2. * Corresponding author: [wangyufei0003@163.com](mailto:wangyufei0003@163.com) (Y. Wang). [↑](#footnote-ref-2)
